# Mediators of the Disparities in Depression Between Sexual Minority and Heterosexual Individuals: A Systematic Review

**DOI:** 10.1007/s10508-020-01862-0

**Published:** 2021-03-10

**Authors:** Angeliki Argyriou, Kimberley A. Goldsmith, Katharine A. Rimes

**Affiliations:** 1 Department of Psychology, Institute of Psychiatry, Psychology and Neuroscience, King’s College London, De Crespigny Park, London, SE5 8AF UK; 2 Department of Biostatistics and Health Informatics, Institute of Psychiatry, Psychology, and Neuroscience, King’s College London, De Crespigny Park, London, UK

**Keywords:** Sexual minority, Sexual orientation, Depression, Mediation, LGB

## Abstract

Evidence suggests that sexual minorities (e.g., those identifying as lesbian, gay, or bisexual) experience increased rates of depression compared to heterosexual individuals. Minority stress theory suggests that this disparity is due to stigma experienced by sexual minorities. Stigma processes are proposed to contribute to reduced coping/support resources and increased vulnerability processes for mental health problems. This review provided a systematic examination of research assessing the evidence for mediating factors that help explain such disparities. A literature search was conducted using the databases PubMed, PsycINFO, and Web of Science. The review included 40 identified studies that examined mediators of sexual minority status and depressive outcomes using a between-group design (i.e., heterosexual versus sexual minority participants). Studies of adolescents and adult samples were both included. The most common findings were consistent with the suggestion that stressors such as victimization, harassment, abuse, and increased stress, as well as lower social and family support, may contribute to differing depression rates in sexual minority compared to heterosexual individuals. Differences in psychological processes such as self-esteem and rumination may also play a role but have had insufficient research attention so far. However, caution is needed because many papers had important methodological shortcomings such as the use of cross-sectional designs, inferior statistical analyses for mediation, or measures that had not been properly validated. Although firm conclusions cannot be drawn, the current evidence base highlights many factors potentially suitable for further exploration in high-quality longitudinal research or randomized studies intervening with the potential mediators.

## Introduction

### Sexual Minorities and Depression

Systematic reviews have reported that compared to heterosexual people, sexual minority individuals (e.g., those identifying as lesbian, gay, or bisexual) have elevated rates of mental health problems and are as much as four times more likely to attempt suicide (King et al., 2008; Plöderl & Tremblay, 2015). A strong link has been consistently demonstrated between sexual minority status and depression in particular (e.g., Bostwick et al., 2010; Chakraborty et al., 2011; Pakula & Shoveller, 2013). A meta-analysis found that the risk of 12-month prevalence of depression in sexual minority individuals was at least twice that of heterosexual controls (King et al., 2008). Similar differences in prevalence rates have been found for heterosexual versus sexual minority youth (e.g., Marshal et al., 2011), suggesting that disparities in depression may appear early in life.

Robust research evidence about the mechanisms through which such disparities come about would be both theoretically and clinically valuable. For example, the identification of intermediate factors that contribute to elevated rates of depression in this population would be instrumental for designing and refining effective prevention programs that would help protect at-risk LGB individuals and developing targeted therapeutic approaches for sexual minority people who experience depression.

Minority stress theory has been one of the main theoretical frameworks used to explain the differences in the rates of depression and other mental health problems between sexual minorities and heterosexuals (Meyer, 2003). According to the theory, being a member of a minority group exposes individuals to discrimination, stigma, and prejudice. Such exposure creates a stressful social environment which contributes to the presence of mental health problems. Meyer suggested that such minority stressors may be distal (external to the person) or proximal, i.e., internal processes about how the individual relates to their identity. Distal stressors include prejudice events such as discrimination and violence, while proximal events include sexual minority-specific internalized stressors such as internalized homophobia, expectations of rejection, and concealment stress. Indeed, evidence indicates that sexual minority individuals face multiple stressors, often starting early in their lives, including peer victimization, physical assault, abuse, and rejection from family and friends (e.g., Balsam et al., 2005; Corliss et al., 2002). There is also a lot of research demonstrating that sexual minority individuals experience a multitude of internal minority stressors such as perceived stigma and expectations of rejection and discrimination, stress about disclosure and concealment, and internalized negative attitudes about their sexual identity (see Meyer, 2003 for a review).

Hatzenbuehler (2009) expanded on minority stress theory by suggesting that the increased stress that sexual minority individuals are exposed to is likely to increase the likelihood of general maladaptive cognitive processes, unhelpful coping and emotion regulation strategies, and reduced social support, all of which may in turn increase the risk for mental health problems. While Meyer’s work focused on the distal and external stressors that sexual minorities experience as well as the sexual minority-specific proximal factors such as internalized homophobia, Hatzenbuehler’s framework shifted the focus to the intermediate cognitive, regulatory, and social mechanisms through which minority stressors lead to mental health problems, including depression. Furthermore, Hatzenbuehler emphasized the importance of examining whether general psychological processes that are known vulnerability factors in the general population are heightened in sexual minorities and whether they can therefore help explain the increased prevalence of mental health problems in sexual minorities compared to heterosexuals.

### Mediation Analysis

In order to understand the intermediate factors that explain the causal relationship between sexual orientation and depression, it is important to look at research that examines mediating variables. Mediation is a process whereby an independent variable is thought to cause change in an intervening variable which in turn causes change in the dependent variable (Lockwood et al., 2002; MacKinnon et al., 2002). In this sense, a hypothesized mediation model will generally constitute a causal chain of events; the plausibility of each of these causal relationships needs to be considered and justified. It follows that a key assumption in mediation analysis is temporal ordering, given that causal relationships are being hypothesized (Cole & Maxwell, 2003). The causal chain described above implies that the independent, mediator, and dependent variables should be measured separately in an ordered fashion in time. Therefore, studies measuring these variables longitudinally are generally considered methodologically superior.

A review of research examining mediators should include the evaluation of the robustness of statistical methods used for mediation analysis. Indeed, several considerations need to be made in assessing the quality of such methodology: Statistical methodology on mediation analysis has developed significantly since the causal steps approach to mediation was developed by Baron and Kenny (1986), including the idea that if mediation is hypothesized, it is still important to do a mediation analysis even in the absence of an effect of the independent on the dependent variable (Emsley et al., 2010; Goldsmith et al., 2018a; MacKinnon & Dwyer, 1993). Mediation analyses now generally focus on a product of coefficients mediated effect (a path x b bath), which can be estimated efficiently in one step using the structural equation modeling (SEM) framework, tests of the joint significance of a and b paths, and the Sobel test of significance of the indirect effect and bootstrapping to calculate mediated effect confidence intervals (Goldsmith et al., 2018b; MacKinnon, 2001; MacKinnon et al., 2004; Sobel, 1982). In addition, in recent years, mediation analysis has focused on sources of bias, such as confounding; researchers should adjust for baseline mediator and outcome measures and include all important potential confounders of the relations in the mediation models (Dunn et al., 2013; Goldsmith et al., 2018a; Imai et al., 2010; Pickles et al., 2015; VanderWeele & Vansteelandt, 2009).

### The Current Study

In recent years, research has investigated factors contributing to the mental health disparities between heterosexual and sexual minority youth and adults by looking at mediators of the relation between sexual orientation and depressive symptomatology. Examining mediators can help us better understand the mechanisms through which both sexual minority status and the stigma associated with it confer risk for depression (Hatzenbuehler, 2009). Moreover, assessing the quality of statistical methodology and design (e.g., temporal ordering of variables) that the literature has used to test mediation is important in drawing conclusions about which mediators are causally contributing to the development of depression in sexual minorities. This would help provide robust evidence for appropriate targets for prevention and intervention that would help end disparities between sexual minorities and their heterosexual peers. No study to date has systematically reviewed between-group studies that use mediation analysis to examine evidence regarding different psychosocial factors that may explain the differences in rates of depression between heterosexual and sexual minority individuals.

Therefore, the aim of the present study is to identify the factors that mediate the relation between sexual minority status and depressive symptoms by systematically reviewing research studies in the literature that use mediational approaches to investigate the disparities among heterosexual and sexual minority individuals. The study also reports the theoretical models used to derive the hypotheses tested in the included studies.

## Method

Prior to data extraction, the review was registered with PROSPERO (registration number CRD42017079383). The review was conducted using PRISMA guidelines for systematic reviews (Moher et al., 2009).

### Data Sources and Search Strategy

A search of published studies was conducted using the following electronic databases: PsycINFO, PubMed, and Web of Science. The search term was: (LGBT OR sexual minorit* OR sexual orientation OR gay OR lesbian OR bisexual OR queer OR homosexual* OR LGB OR non-heterosexual) AND (Heterosexual* OR non-minority) AND (depress* OR mood) AND (mechanism* OR mediat* OR predict* OR factor* OR explain OR caus* or risk factor or structural equation model*). Additional studies were retrieved by cross-referencing of selected articles, and through hand searches. The literature search was completed on October 27, 2017, and was updated on October 21, 2019.

### Inclusion and Exclusion Criteria

We included studies that: (1) were published in peer reviewed journals; (2) included a statistical group comparison between heterosexual and sexual minority status individuals; (3) used a measure of depressive symptoms or a diagnosis of depression as an outcome variable; (4) used analyses that tested hypothesized mediation effects with sexual orientation as the independent variable and depression as the dependent variable. We excluded studies that: (1) were non-empirical (reviews or theory papers); (2) did not have the full description of the study available (e.g., conference abstracts); (3) were published in languages other than English. We did not exclude studies based on publication year, sample size, age groups used, or whether they used a subsample of the population of interest. An initial screening of all title and abstracts returned using the aforementioned search strategy was conducted by the first author. A second independent reviewer also screened a random 10% of the titles and abstracts returned. Studies that met the eligibility criteria based on the initial screening were screened using the full-text papers by the first author and a subgroup were also screened by an independent reviewer. The kappa statistic was used to measure inter-rater agreement.

### Data Extraction

The following data were extracted from included studies: study title; authors; year; design (cross sectional or longitudinal); country/setting; population/sample characteristics; recruitment strategy; total and group sample size; sexual orientation measure; hypothesized mediator(s); measure(s) for mediator(s); depression measure; confounders; type of mediation analysis (series of regression or SEM); test of significance for mediation; statistical analysis details; main findings; and limitations. As studies were methodologically and statistically heterogeneous, a meta-analysis or other methods of statistical pooling to synthesize the findings were not appropriate. The theoretical models, methodology, results, and limitations of the studies are therefore qualitatively summarized in the Results and Discussion sections, with much supporting detail provided in the tables and the Appendix. This process was conducted mainly by authors AA and KAR, with statistical expertise provided by KAG.

### Quality Assessment

A quality assessment measure developed for treatment mediation studies by Lubans et al. (2008) and expanded in other studies (Cerin et al., 2009; Lee et al., 2015; Mansell et al., 2013) was further adapted for the purposes of this study. This included four additional items being added from the Quality Assessment Tool for Observational Cohort and Cross-Sectional Studies (US Department of Health and Human Services, 2014) and the Quality Assessment Tool for Quantitative Studies (Effective Public Health Practice Project, 2009) to address the methodological quality of the predictor, sampling procedures, representativeness, and response/uptake. The quality assessment focused on the mediation hypotheses of the studies that were relevant to this review. A score for each study was computed by assigning a value of 0 (no) or 1 (yes) to each of 12 questions listed in Table 1. If a study did not explicitly report information related to an item, it was assigned 0 for that item. A total score was calculated by summing the scores of the 12 items for each of the studies. Studies which scored 0–4 were classified as of *poor* quality, 5–8 were classified as of *fair* quality, and 9–12 were classified as of *good* quality. For item 6 (statistically appropriate/acceptable data analysis methods), studies were assigned 1 if they conducted and reported a test of significance for the mediated effect either through testing of the product of coefficients (e.g., Sobel test, bootstrapping) or joint testing of the a and b paths, as recommended by MacKinnon et al. (2002). Studies were assigned a 0 if they solely used the causal steps approach (Baron & Kenny, 1986) or other approaches to mediation such as SEM without testing for statistical significance of the indirect effect. Quality assessment ratings were done by two raters. Inter-rater reliability was substantial (*κ* = .782), and discrepancies were resolved through discussion.

**Table 1:** Quality assessment tool

Item:	Score: 0/1
1. Did the study cite a theoretical framework?	
2. Was the independent variable clearly defined, valid (face validity), and reliable, and implemented consistently across participants?	
3. Were the psychometric characteristics of the mediator variable reported and were they within accepted ranges? (Computed from the present study or a reference provided)	
4. Were the psychometric characteristics of the depression variable reported and were they within accepted ranges? (Computed from the present study or a reference provided)	
5. Did the study report a power calculation? If so, was the study adequately powered to detect mediation?	
6. Were statistically appropriate/acceptable methods of data analysis used?^a^	
7. Did the study ascertain whether changes in the mediating variable preceded changes in the outcome variable?	
8. Did the study ascertain whether changes in the predictor variable preceded changes in the mediator variable?	
9. Did the study control for possible confounding factors?	
10. Were all the subjects selected or recruited from the same or similar populations (including the same time period)? Were inclusion and exclusion criteria for being in the study prespecified and applied uniformly to all participants?	
11. Are the individuals selected to participate in the study likely to be representative of the LGB and heterosexual population?	
12. (a) Was 80% or more of potential participants included at point of relevant analyses? (b) If the study was longitudinal, was loss to follow-up after baseline 20% or less?	

^a^Studies were assigned 1 if they conducted and reported a test of significance for the mediated effect either through testing of the product of coefficients (e.g., Sobel test, bootstrapping) or joint testing of the a and b paths (MacKinnon et al., 2002). Studies were assigned a 0 if they solely used the causal steps approach (Baron & Kenny, 1986) or other approaches to mediation without testing for statistical significance of the indirect effect

## Results

### Included Studies

**Fig. 1:**
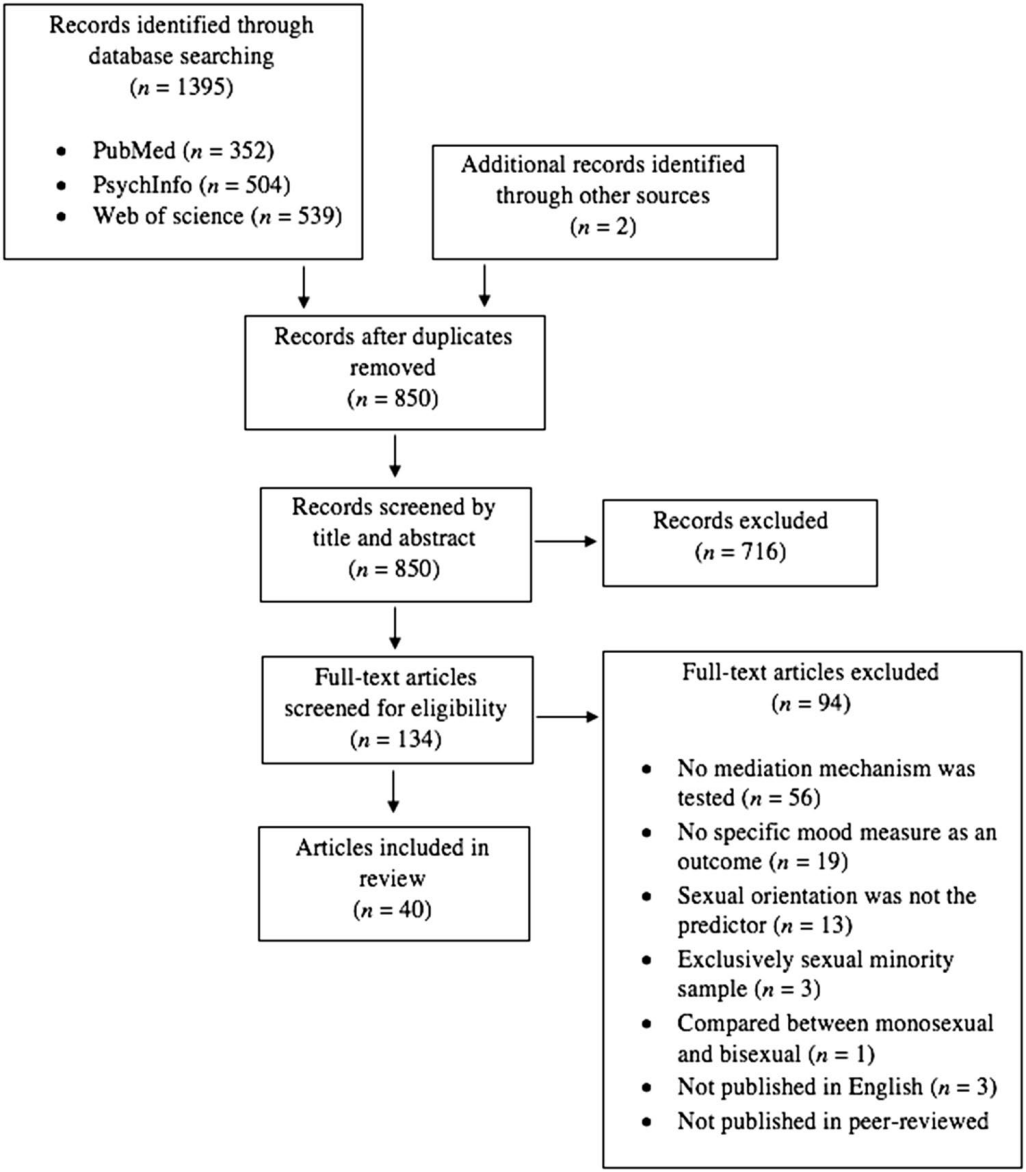
Study flow diagram

The searches identified 1397 studies, 547 of which were duplicates. Of the remaining 850 studies, 716 were excluded based on the title or the abstract when it was evident that they either did not meet the inclusion criteria or at least one of the exclusion criteria. Information about the relevant inclusion and exclusion criteria for the studies excluded in the first stage of the screening was not recorded. The number of full-text articles assessed for eligibility was 134. Inter-rater agreement about decisions to include studies or not was very good, *κ* = .939 (95% CI 0.87, 1.00). Discrepancies were resolved through discussion relevant to the inclusion and exclusion criteria. The final number of studies meeting the inclusion and exclusion criteria and therefore included in the review was 40. Figure 1 illustrates the flow of studies.

### Quality Assessment

The results of the quality assessment are shown in Table 2. Most studies were rated as having *fair* methodological quality. Five studies were rated as being of *good* quality and seven studies rated as being of *poor* methodological quality.

**Table 2:** Quality assessment

	1. Theory	2. Predictor	3. Mediator	4. Outcome	5. Power	6. Analysis	7. Mediator before outcome	8. Predictor before mediator	9. Confounders	10. Recruitment	11. Representativeness	12. Response uptake	Quality rating
1. Almeida et al. (2009)	1	1	0	1	0	1	0	0	0	1	1	0	Fair
2. Burns et al. (2016)	1	1	0	0	0	0	0	1	1	1	1	1	Fair
3. Burton et al. (2013)	1	1	1	1	0	1	0	1	1	1	0	1	Good
4. Donahue et al. (2017)	1	1	0	0	0	0	0	0	1	1	0	0	Poor
5. Frisell et al. (2009)	1	0	0	1	0	0	0	0	1	0	0	0	Poor
6. Frost and LeBlanc (2014)	1	0	1	1	0	1	0	0	1	1	0	0	Fair
7. Hatzenbuehler et al. (2008)	1	1	1	1	0	1	1	0	1	1	0	0	Fair
8. Hatzenbuehler et al. (2012)	1	1	0	1	1	0	0	0	1	1	1	0	Fair
9. Hughes et al. (2014)	1	1	0	0	0	0	0	0	1	0	0	0	Poor
10. Krueger et al. 2018	1	1	0	0	0	1	0	0	1	1	0	1	Fair
11.la Roi et al. (2016)	1	1	1	1	0	1	1	0	1	1	1	0	Good
12. Luk et al. (2018)	1	1	0	1	0	1	1	0	1	1	1	1	Good
13. Luk et al. (2019)	1	1	1	1	0	1	0	1	1	1	1	1	Good
14. Martin-Storey and August (2016)	1	1	1	1	0	1	0	0	1	0	0	1	Fair
15. Martin-Storey and Crosnoe (2012)	1	1	1	1	0	1	0	0	1	1	1	0	Fair
16. McLaren (2008)	1	1	1	1	0	0	0	0	1	0	0	0	Fair
17. McLaren et al. (2007)	1	1	1	1	0	0	0	0	1	0	0	1	Fair
18. McLaughlin et al. (2012)	1	1	0	1	0	1	0	0	1	1	1	0	Fair
19. McNair et al. (2005)	1	1	0	0	0	0	0	0	1	1	1	0	Fair
20. Mereish et al. (2019)	1	1	0	1	0	1	0	0	1	1	0	0	Fair
21. Miller and Irvin (2016)	1	0	0	0	0	1	0	0	1	1	0	0	Poor
22. Needham and Austin (2010)	1	1	1	1	0	0	0	0	1	1	1	0	Fair
23. Oginni et al. 2018	1	1	0	0	1	0	0	0	1	1	0	1	Fair
24. Pakula et al. (2016)	1	1	0	0	0	1	0	0	1	1	1	0	Fair
25. Pearson and Wilkinson (2013)	1	1	1	1	0	1	1	0	1	1	1	1	Good
26. Przedworski et al. (2015)	1	1	1	1	0	0	0	0	1	1	0	1	Fair
27. Riley et al. (2016)	1	1	1	1	0	1	1	0	1	1	0	0	Fair
28. Robinson et al. (2013)	1	1	0	1	0	1	1	0	1	1	1	0	Fair
29. Rosario et al. (2014)	1	1	1	1	1	0	1	0	1	1	0	0	Fair
30. Safren and Heimberg (1999)	1	1	1	1	0	0	0	0	0	0	0	0	Poor
31. Shenkman et al. (2019)	1	1	1	1	0	1	0	0	1	1	0	0	Fair
32. Sigurvinsdottir and Ullman (2016)	1	1	1	1	0	1	0	0	1	0	0	0	Fair
33. Smith et al. (2016)	1	0	0	1	0	0	0	0	0	1	0	0	Poor
34. Spencer and Patrick (2009)	1	1	1	1	1	0	0	0	1	0	0	1	Fair
35. Szalacha et al. (2017)	1	1	0	1	0	0	0	0	1	1	1	0	Fair
36. Tate and Patterson (2019)	1	1	1	1	1	1	0	0	1	1	1	0	Good
37. Teasdale and Bradley-Engen (2010)	1	0	0	1	0	0	1	0	1	1	1	0	Fair
38. Ueno (2010)	1	0	1	1	0	0	0	0	1	0	0	0	Poor
39. Wong et al. (2017)	1	1	0	1	0	1	0	0	1	1	0	0	Fair
40. Woodford et al. (2014)	1	1	0	1	0	1	0	0	1	1	0	0	Fair

### Study Characteristics

Study characteristics are summarized in Table 3. Of the 40 studies, 28 had a cross-sectional design and 12 had a longitudinal design. The longitudinal studies either measured sexual orientation and mediator at time 1 and depression at time 2 or sexual orientation at time 1 and mediator and depression at time 2, with none of the studies collecting measures of the three variables at three different time points. Most of the studies took place in the U.S. (*n* = 26), while some took place in Australia (*n* = 5), Sweden (*n* = 2), the UK (*n* = 1), the Netherlands (*n* = 1), China (*n* = 1), Canada (*n* = 1), Israel (*n* = 1), and Nigeria (*n* = 1). One study took place in both the U.S. and Canada.

**Table 3:** Study design, sample, setting, and mediators tested

Study	Design	Sample	Setting/country	Mediators tested
1. Almeida et al. (2009)	Cross sectional	*n* = 1032 103 LGB, 929 non-LGB	Public high schools Boston, USA	Perceived discrimination
2. Burns et al. (2016)	Longitudinal	*n* = 4824 149 LGB, 4675 non-LGB	Community sample of adults Australia	Major life events Social support Health and behaviors Behavioral activation and inhibition
3. Burton et al. (2013)	Longitudinal	*n* = 197 55 LGB, 137 non-LGB	Adolescent medicine clinics Pennsylvania and Ohio, USA	Sexual minority specific victimization
4. Donahue et al. (2017)	Cross sectional	*n* = 3987 331 LGB, 3656 non-LGB	Population-based sample of adolescent twins Sweden	Victimization
5. Frisell et al. (2009)	Cross sectional	*n* = 16,728 1241 had same-sex partners, 15,487 did not have same-sex partners	Population-based sample of adult twins Sweden	Perceived victimization Hate crime victimization
6. Frost and LeBlanc (2014)	Cross sectional	*n* = 431 239 LGB, 192 non-LGB	Online study of adults USA and Canada	Nonevent stress
7. Hatzenbuehler et al. (2008)	Longitudinal	*n* = 1071 29 LGB, 1042 non-LGB	Middle schools Connecticut, USA	Emotional regulation: emotional awareness and rumination
8. Hatzenbuehler et al. (2012)	Cross sectional	*n* = 14,319 151 LG, 708 BI, 13,353 non-LGB	Nationally representative sample of adolescents USA	Social isolation Degree of connectedness Social status
9. Hughes et al. (2014)	Cross sectional	*n* = 1573 326 L, 124 ML, 27 BI 72 MH, 1573 non-LGB	Women from two large studies (national & Chicago Metropolitan area) USA	Victimization
10. Krueger et al. (2018)	Cross sectional	*n* = 14,216 11,756 concordant H^a^, 539 LGB, 1406 MH, 515 discordant^a^ H	Nationally representative sample of young adults USA	Perceived stress
11. la Roi et al. (2016)	Longitudinal	*n* = 1738 151 LGB, 1587 non-LGB	Large cohort study of adolescents Five municipalities in the north of Netherlands (urban and rural)	Peer victimization Parental rejection
12. Luk et al. (2018)	Longitudinal	*n* = 2396 99 LGB, 2080 non-LGB	Nationally representative sample of adolescents and young adults USA	Family satisfaction Peer support Cyberbullying victimization Unmet medical needs
13. Luk et al. (2019)	Longitudinal	*n* = 2012 1839 H, 37 LG, 104 BI, 32 Q	National cohort study of adolescents USA	Cyber behaviors (weekday time spent on cyber behavior, weekend time spent on cyber behavior, social media use)
14. Martin-Storey and August (2016)	Cross sectional	*n* = 251 93 LGB, 158 non-LGB	University and college students Southwestern city, USA	Harassment due to gender nonconformity Harassment due to sexual minority status
15. Martin-Storey and Crosnoe (2012)	Cross sectional	*n* = 957 40 LGB, 917 non-LGB	Multi-site study of adolescents USA	Harassment due to sexual minoritystatus Self-concept Self-regulation Friendship quality Parental support Quality of the school environment
16. McLaren (2008)	Cross sectional	*n* = 386 184 L, 202 non-LGB	Community sample of women Victoria, Australia (urban, rural, regional areas)	Sense of belonging
17. McLaren et al. (2007)	Cross sectional	*n* = 273 137 G, 136 non-LGB	Community sample of men Australia	Sense of belonging
18. McLaughlin et al. (2012)	Cross sectional	*n* = 13,962 227 LG, 245 BI, 13,490 non-LGB	National cohort study of adolescents/young adults USA	Exposure to adversity
19. McNair et al. (2005)	Cross sectional	*n* = 19,559 *Younger cohort*: *n* = 9260 92 L, 75 BI, 634 MH, 8482 non-LB *Mid-age cohort:* *n* = 10,299 126 L, 16 BI, 122 MH, 10,035 non-LB	Large national sample of women Australia	Stress Abuse Social support
20. Mereish et al. (2019)	Cross sectional	*n* = 1129 839 H, 224 MH, 66 LGB	Children and adolescents in a large county in North Carolina USA	Cyber victimization Bias-based victimization
21. Miller and Irvin (2016)	Cross sectional	*n* = 4769 95 LGB, 4674 non-LGB	Nationally representative sample of intimate partner violence survivors USA	Type of victimization Emotional support
22. Needham and Austin (2010)	Cross sectional (baseline data as confounder)	*n* = 11,195 193 LG, 192 BI, 10,768 non-LGB	Nationally representative sample of adolescents and young adults USA	Parental support
23. Oginni et al. (2018)	Cross sectional	*n* = 162 81 Gay, 81 H	University sample South-Western Nigeria	Family-related variables Resilience
24. Pakula et al. (2016)	Cross sectional	*n* = 222,548 2893 LG, 2225 BI, 217,652 non-LGB	Large national multi-year sample of adults Canada	Perceived life stress
25. Pearson and Wilkinson (2013)	Longitudinal	*n* = 11,601 770 LGB, 10,831 non-LGB	Nationally representative sample of adolescents USA	Family relationships: Perceived parental closeness Parental involvement Perceived family support
26. Przedworski et al. (2015)	Cross sectional	*n* = 4673 232 LGB, 4441 H	National study of medical students USA	Social stressors
27. Riley et al. (2016)	Longitudinal	*n* = 1777 75 LGB, 1702 H	First year university students USA	Stress Coping styles
28. Robinson et al. (2013)	Longitudinal	*n* = 4135 187 LGB, 3948 H	Nationally representative sample of young people UK	Victimization
29. Rosario et al. (2014)	Longitudinal	*n* = 6122 101, 101 BI, 611 MH, 5309 H	Cohort study of early adolescent children USA	Attachment Parental affection
30. Safren and Heimberg (1999)	Cross sectional	*n* = 104 56 LGB, 48 non-LGB	Community sample of youth Philadelphia USA	Social support Coping Stress
31. Sigurvinsdottir and Ullman (2016)	Longitudinal	*n* = 905 95 BI, 810 non-LGB	Community sample of bisexual and heterosexual sexual assault women survivors Chicago metropolitan area, USA	Perceived social support Frequency of social contact
32. Smith et al. (2016)	Cross sectional	*n* = 299 29 LGB, 270 non-LGB	Undergraduate psychology students in a large public university Pacific Northwest, USA	Institutional betrayal
33. Shenkman et al. (2019)	Cross sectional	*n* = 795 445 H, 350 LG	Online convenience/targeted sample Israel	Attachment avoidance
34. Spencer and Patrick (2009)	Cross sectional	*n* = 306 66 LG, 24 BI	Online convenience sample of young adults USA	Social support Personal mastery
35. Szalacha et al. (2017)	Cross sectional	*n* = 8850 568 MH, 100 BI, 99 L, 8083 non-LB	National study of women Australia	Interpersonal violence
36. Tate and Patterson (2019)	Cross sectional	*n* = 15,701 14,973 H and MH, 248 BI, 340 LG	Large national sample of young adults	Mother relationship quality Father relationship quality Perceived stress
37. Teasdale and Bradley-Engen (2010)	Longitudinal	*n* = 11,243 787 LGB, 10,456 non-LGB	Large national sample of adolescents USA	Social stress Social support
38. Ueno (2010)	Cross sectional	*n* = 1492 64 had same-sex contact, 1428 did not have same-sex contact	Community sample of young adults Miami-Dade, USA	Victimization Major discrimination events Daily discrimination Negative life events Chronic strains Family support Friend support Optimism Mastery Self-esteem Mattering Fun-seeking orientation Relationship status Number of sexual relationships Early first sex Parents’ permissiveness of drug use Friends’ permissiveness of drug use Friends’ drug use
39. Wong et al. (2017)	Cross sectional	*n* = 1076 142 LGB, 934 H	Multi-site university sample China	Dating violence Sexual orientation concealment
40. Woodford et al. (2014)	Cross sectional	*n* = 2428 426 LGB, 2002 H	University students Midwest, USA	Interpersonal mistreatment

LGB, lesbian, gay, bisexual; L, lesbian; ML, mostly lesbian; G, gay; BI, bisexual MH mostly heterosexual; H, heterosexual; Q, questioning

^a^Concordant H identified as heterosexual and their reported attractions and behaviors were all toward the opposite sex. Discordant H identified as heterosexual but reported same-sex attractions and/or behaviors

### Population

Information about the sample is provided in Table 3 with additional information in the Appendix. Although a few studies did not provide a specific age range, of the 40 studies, 13 seemed to have had predominantly early to late adolescent samples (11 to 19 years), 14 had young adult or university student samples (17 to 29 years), 11 used exclusively adult samples (18 years and over), while one study used both a young adult and an older adult cohort and another study used a young adult and mid-adult sample. Some of the studies used subsamples of the population, such as sexual assault survivors, victims of intimate partner violence, or exclusively Black American participants. Some studies used other samples that may limit the generalizability of their findings, including samples of twin siblings, children of registered nurses, medical students, and undergraduate psychology students. Several studies used the same or overlapping samples, and it is therefore not possible to report an overall number of participants investigated across the included papers.

### Theoretical Framework

Many of the studies derived their research questions from broader theoretical frameworks relating to sexual minority individuals’ increased exposure to social stress, with the most often-cited theory being minority stress theory (Meyer, 2003). A few studies cited Hatzenbuehler’s (2009) psychological mediation framework that includes more general mechanisms through which exposure to social stressors renders sexual minorities more vulnerable to mental health problems. Similarly, other studies explored general psychosocial processes that have been established as risk factors in the general population and sought to explore their specific associations with sexual minority identity. Specific hypotheses pertaining to the sexual orientation disparities in mental health problems investigated in other studies included: the role of unmeasured genetic and shared environmental factors; the differential incidence and impact of sexual and physical violence in sexual minority populations; the interacting role of gender/sex and sexual orientation; childhood adversity; cultural-specific factors associated with concealment of sexual orientation; theory of human relatedness and social belonging (Hagerty et al., 1993); and the importance of family support and attachment for this population.

### Measurement of Sexuality

Sexuality was assessed in a number of ways. Most studies used sexual identity or sexual orientation questions and response options. Seven studies asked about sexual or romantic attraction, and one asked about preference for romantic partners. One study asked about the gender of individuals with whom participants were in relationship and two studies inquired about the number of the same-sex and the opposite-sex people respondents had sexual intercourse with. A study asked both about identity and behavior, while another study averaged the responses from three items asking about fantasies, attraction, and behavior. One of the studies asked about identity but encompassed behavioral indicators in the response options (e.g., homosexual with some heterosexual experience). Two studies did not report how they assessed sexuality.

Sexuality-related response options available also varied greatly with studies using from three to seven categories of sexuality, and one study using a fill-in-blank response. Most studies categorized sexual minority and heterosexual individuals into two groups, with some citing power concerns as the reason they did not distinguish among more sexual minority groups. Studies often included response categories such as mostly homosexual, mostly heterosexual, other, and questioning, but they varied on how they later treated these responses. For example, while some of the studies included participants who selected mostly heterosexual in the sexual minority group, two studies categorized them as a distinct group, another study placed them in the heterosexual group despite having a bisexual category in their analysis, and another study excluded them from the analysis. Similarly, participants who chose other were either placed in the sexual minority group or were excluded from the analysis. Individuals attracted to neither males or females, not sure, and with no sexual experience were excluded from the analyses, while two studies that included a questioning response, placed the participants selecting it in the sexual minority group. Finally, three studies excluded participants who identified as bisexual from their analyses.

### Outcome Measures

Most studies used validated self-report measures of depressive symptoms including the Center for Epidemiological Studies Depression (Kohout et al., 1993; Radloff, 1977); the Beck Depression Inventory II (Beck et al., 1996); the Depression Anxiety Stress Scales (Lovibond & Lovibond, 1995); the Modified Depression Scale (Orpinas, 1993); the Youth and Adult Self-Report (Achenbach & Rescorla, 2001, 2003); the Zung Depression Scale (Zung, 1965); the Hospital Anxiety and Depression Scale (Chinese version; Leung et al., 1993); the Brief Symptom Inventory (Derogatis, 1993); the Goldberg Depression & Anxiety Scale (Goldberg et al., 1988); the PROMIS Emotional Distress-Depression scale (Pilkonis et al., 2011); and the Children’s Depression Inventory (Kovaks, 1992). Two studies used items the authors validated as a scale or as a latent variable for the purposes of their study. One study used diagnostic interview questions to code participants as having or not having depression. Some studies asked questions that had not been validated regarding the presence of a diagnosis of depression and then used them to classify participants as having or not having depression. One study used both a self-report measure and questions about history of depression as outcomes, while another study used both the Structured Clinical Interview (SCID) and a self-report measure. Finally, one study used a single-item depression measure as well as a validated questionnaire.

### Mediators

Mediators investigated can be found in Table 3, while details about the measures used for the mediators can be found in the Appendix. Only three studies assessed the independent variable at an earlier time point than the mediator, and eight studies assessed the mediator before the outcome. Half of the studies used measures of the proposed mediators for which evidence of validity and reliability was limited or not provided. Most studies used one or two mediators in their analysis, while others analyzed multiple mediators. A few of the studies used a mediator assessed by a single question not associated with a validated scale. Included studies used a variety of variables as hypothesized mediators of the association between sexual minority status and depression.

Many studies looked at self-reports of victimization, harassment, discrimination, or abuse as mediators, either relating to sexual minority status or more generally. Other, more general, stress-related mediators included major life events or chronic stress. Some studies investigated sex, relationship, friendship, or family-related mediators. Other social factors tested included social support, the quality of the school environment, sense of belonging, and institutional betrayal. Intrapersonal factors investigated as mediators included emotional regulation, self-regulation, coping styles, resilience, self-concept, and master*y*.

### Confounders

The overwhelming majority of studies controlled for some confounders with most studies controlling for sociodemographic variables (e.g., age, sex or gender, ethnicity/race, place of residence, education, income, family structure, relationship status). Only a few studies controlled for baseline levels of depression. A few studies also controlled for familial confounding (confounding caused by shared environmental/genetic risk factors) by comparing participants to siblings or controlling for parental psychopathology. One of the studies included history of adverse childhood experiences as a confounder, while three studies controlled for violence and victimization. One study controlled for social desirability, and another study that used different recruitment methods included recruitment method as a confounder. A minority of studies did not use any confounders.

### Statistical Analysis

The statistical approaches undertaken in the papers are shown in Table 4. Only five studies reported power calculations. With regard to data analytic approaches, many of the studies followed mediation procedures similar to the causal steps approach proposed by Baron and Kenny (1986), while some of the studies used SEM. Many of the studies did not conduct a test for the mediated effect either through testing the significance of the product of coefficients estimate of the indirect effect, or joint testing of the a and b paths.

**Table 4:** Statistical analysis and findings

Study	Statistical analysis	Findings^a^
1. Almeida et al. (2009)	Series of regressions Sobel test	Perceived discrimination mediated the relation between sexual minority status and depressive symptoms. The mediation was especially pronounced for boys
2. Burns et al. (2016)	Series of regressions	A bisexual but not a homosexual orientation was found to predict increased rates of depression compared to those with a heterosexual orientation. This association was no longer significant when other significant predictors were included in the model, including social support, physical health, smoking status, and history of sexual trauma, suggesting the potential mediating role of these variables (although the authors did not describe these as mediators)
3. Burton et al. (2013)	Series of regressions Product of coefficients: bootstrapping	Sexual minority-specific victimization mediated the effect of reported sexual minority status and depressive symptoms, controlling for baseline depressive symptoms and demographic variables
4. Donahue et al. (2017)	Series of regressions	Results suggested that victimization attenuated the relation between sexual minority status and depression. This possible mediation effect was decreased when controlling for unmeasured familial confounding by comparing sexual minority youth to their heterosexual same sex twin siblings
5. Frisell et al. (2009)	Series of regressions	Adjusting for perceived discrimination and hate crime victimization reduced the relation between same-sex sexual experience and depressive symptoms, suggesting evidence for mediation (although the authors did not describe the variables as mediators). When controlling for familial confounding with the use of within-twin-pair comparisons, men with same-sex contact and those without did not differ in depression rates. For women, a significant difference based on same-sex contact remained, which disappeared when accounting for perceived discrimination and hate crime victimization
6. Frost and LeBlanc (2014)	Series of regressions Bootstrapping	Controlling for demographic variables, greater nonevent stress (i.e., barriers to life pursuits in relationships and work) mediated the relation between sexual orientation and depression symptoms
7. Hatzenbuehler et al. (2008)	SEM Sobel test	Greater rumination and poorer emotional awareness mediated the association between same-sex attraction and depressive symptoms, while controlling for baseline levels of depression
8. Hatzenbuehler et al. (2012)	Series of regressions	Controlling for demographic factors, violence, and victimization, sexual minority status was no longer significantly associated with depression in boys when social isolation was included in the model. No mediation hypotheses were tested for girls, as social network variables were not found to be associated with depression in girls
9. Hughes et al. (2014)	Series of regressions	Controlling for demographic variables and parental drinking, no differences in depression were found between heterosexual and lesbian women. However, bisexual women were found to have increased rates of depression compared to heterosexual women. After adjusting for the number of types of victimization, the difference in depression between bisexual and heterosexual women was no longer statistically significant
10. Krueger et al. (2018)	SEM Unspecified test of significance	Perceived stress mediated the association between sexual minority status and depressive symptoms for all sexual minority groups of women when compared to heterosexuals. However, perceived stress was only related to sexual minority status for mostly heterosexual men and not gay/bisexual or discordant heterosexual men when compared to heterosexuals
11. la Roi et al. (2016)	SEM: latent growth modeling Product of coefficients	Disparities in depression between sexual minority girls and youth of bisexual identity, present since age 11, were mediated by both victimization and parental rejection. Depression differences in boys were not found. However, peer victimization but not parental rejection mediated the association between sexual minority status and depressive symptoms for boys too. Both peer victimization and parental rejection mediated the association between bisexual identity and depressive symptoms
12. Luk et al. (2018)	SEM: latent growth modeling Bootstrapping	Family satisfaction, cyberbullying victimization, and unmet medical needs all mediated the relation between sexual minority status and depressive symptoms. Peer support was not found to mediate the association as it was not associated with sexual minority status
13. Luk et al. (2019)	SEM Bootstrapping	Controlling for ethnicity and family affluence, bisexual attraction in adolescence was both directly and indirectly associated with higher depressive symptoms during young adulthood through increased time spent on cyber behaviors (weekday and weekend) and social media. These mediation associations were not found when comparing to gay/lesbian and questioning groups to heterosexuals as these groups did not differ in cyber behaviors and social media use compared to heterosexual youth. Bisexual and questioning females reported higher depressive symptoms than heterosexual females, but such disparities were not found when comparing lesbian youth to heterosexual, or among sexual orientation subgroups in males
14. Martin-Storey and August (2016)	SEM Bootstrapping	Controlling for socioeconomic status and method of recruitment, the relation between sexual orientation and depressive symptoms was mediated by harassment due to gender nonconformity
15. Martin-Storey and Crosnoe (2012)	SEM Delta method	Controlling for demographic variables, baseline depression, and maternal depression, harassment due to sexual minority status mediated the association between sexual minority status and depression. Harassment due to sexual minority status was associated with depression via lowered sense of self-concept and negative perceptions of the school environment
16. McLaren (2008)	Series of regressions	Controlling for demographic variables, results provide some evidence for mediation of lower sense of belonging in the relations between of sexual orientation and dysphoria in women
17. McLaren et al. (2007)	Series of regressions	Controlling for demographic variables, results provide some evidence for mediation of lower sense of belonging in the association between sexual orientation and dysphoria in men
18. McLaughlin et al. (2012)	Series of regressions Sobel test	Controlling for demographic variables, exposure to early life adversity was a significant mediator of the association between gay and lesbian orientation and depression
19. McNair et al. (2005)	Series of regressions	Controlling for demographic variables, results suggested that for the younger cohort, all sexual minority women (mainly heterosexual, bisexual, and exclusively/mainly homosexual) had higher rates of depression than heterosexual women and that stress, abuse, and lower social support attenuated these associations. For the older cohort of women, only the mainly heterosexual group had higher depression rates compared to the heterosexual group, and this difference disappeared when stress, abuse, and lower social support were added to the model
20. Mereish et al. (2019)	Series of regressions Bootstrapping	Controlling for demographic variables, cyber and bias-based victimization mediated the relation between sexual orientation and depression outcomes in Black American young people
21. Miller and Irvin (2016)	Series of regressions Sobel test	Controlling for demographic variables, lower emotional support mediated the relation between sexual orientation and depression for victims of intimate partner violence. The type of abuse experienced (verbal, physical, and sexual) did not mediate the relation
22. Needham and Austin (2010)	Series of regressions	Bisexual women but not lesbian women had elevated depressive symptoms compared to heterosexual women. Controlling for demographic variables, results were consistent with the suggestion that the association between bisexual identity and depressive symptoms was attenuated when parental support was included in the model. Gay and bisexual men were not found to differ to heterosexual men in depression rates
23. Oginni et al. (2018)	Series of regressions	The family-related variables examined resulted in an attenuation in the relation between sexual orientation and depressive symptoms, but this attenuation was not significant. Entering resilience in the model resulted in a significant attenuation in the relations between sexual orientation and depressive symptoms, suggesting the mediating role of resilience (although the authors did not describe it as a mediator)
24. Pakula et al. (2016)	Series of regressions Product of coefficients: bootstrapping	After controlling for demographic variables, greater life stress significantly mediated the associations between sexual identity and mood disorders for both gay/lesbian and bisexual respondents
25. Pearson and Wilkinson (2013)	Series of regressions Sobel test	For girls, perceived closeness with parents and family support mediated the association between same-sex attraction and depressive symptoms. For boys, perceived parental closeness mediated the association of same-sex attraction and depressive symptoms. Results suggested that poorer family relationships were a stronger mediator for girls than for boys
26. Przedworski et al. (2015)	Series of regressions	After controlling for demographic variables, results suggested that social stressors decreased the magnitude of the association between sexual minority status and depression
27. Riley et al. (2016)	Series of regressions Bootstrapping	After controlling for demographic and baseline levels of depression, stress and coping styles (denial, blame, reframing and religion) were not found to mediate the association between sexual identity and depression
28. Robinson et al. (2013)	SEM Unspecified test of significance	In both girls and boys, peer victimization mediated the disparities in indicators of depressive distress
29. Rosario et al. (2014)	Series of regressions	After controlling for demographic variables and sibling clustering, less secure maternal attachment attenuated the relation between sexual orientation and depressive symptoms for bisexual and mostly heterosexual youth compared to heterosexual youth. For lesbian and gay youth, the association disappeared once attachment was entered in the model. There was no evidence that maternal affection mediated depression disparities between the sexual minority subgroups and heterosexuals
30. Safren and Heimberg (1999)	Series of regressions	Sexual minority status was related to potential mediators (although the authors did not describe these as mediators) stress and social support, but not acceptance coping. Sexual minority status was related to depression in a univariate model, but this was no longer the case when the stress, social support, and acceptance coping variables were added into the model
31. Sigurvinsdottir and Ullman (2016)	Series of regressions Sobel test	Heterosexual women survivors of sexual assault had lower depressive symptoms than bisexual women survivors. Lower perceived social support mediated the association between sexual orientation and depressive symptoms
32. Smith et al. (2016)	Series of regressions	Results suggested that greater self-reported institutional betrayal attenuated the relation between sexual minority status and depression
33. Spencer and Patrick (2009)	Series of regressions	The association between sexual orientation and depressive symptoms disappeared when personal resources of social support and mastery were entered into the model. Both social support and personal mastery uniquely contributed to depression variance
34. Shenkman et al. (2019)	Series of regressions Bootstrapping	Controlling for demographic variables, attachment avoidance mediated the association between being gay or lesbian and depressive symptoms
35. Szalacha et al. (2017)	Series of regressions	Having a lesbian or bisexual sexual identity was not found to predict depression, while a mainly heterosexual sexual identity was. Despite the number of types of interpersonal violence emerging as the strongest predictor of depression in the model, no evidence for mediation was found
36. Tate and Patterson (2019)	SEM Bootstrapping	Controlling for sociodemographic variables, higher perceived stress and lower relationship quality with fathers mediated the relation between lesbian, gay, and bisexual identities and depressive symptoms. Lower relationship quality with mothers and higher perceived stress mediated the relation between sexual minority status and depressive symptoms in women but not in men. For men, there was no difference in relationship quality with mothers among sexual orientation groups
37. Teasdale and Bradley-Engen (2010)	Series of regressions	Controlling for demographic variables, results suggested that greater social stress (including victimization, witness victimization, forced sexual encounters, and suicide of a friend) and lower social support (perceived care and social acceptance by peers, parents, and teachers) attenuated the relation between sexual minority status and depressive outcomes
38. Ueno (2010)	Series of regressions	Victimization and daily discrimination attenuated the relation between same-sex contact and depressive symptoms. Negative life events and chronic strain also attenuated the association independently. Similarly, family relationships decreased the association, as well as psychological resources (mastery, self-esteem, and mattering). When all the hypothesized mediators were simultaneously entered in the model the difference between those with same-sex contact and those without was greatly reduced but was still significant. There was no evidence that self-exploratory attitudes (fun-seeking orientation, number of sexual partners, and early sexual initiation) explained the association between same-sex contact and depressive symptoms. The variables of major discrimination, friend support, relationship status, and optimism were not tested for mediation as there were no differences between the groups on these factors
39. Wong et al. (2017)	SEM Bootstrapping and Sobel test	After controlling for demographic variables and adverse childhood experiences, dating violence and sexual orientation concealment both independently mediated the relation between sexual minority status and depressive outcomes
40. Woodford et al. (2014)	Series of regressionsBootstrapping	After controlling for demographic variables, more experiences of interpersonal mistreatment (incivility and heterosexist harassment) mediated the relation between sexual minority status and depression

^a^Terms such as “boys,” “girls,” “men,” and “women” are used to report the findings of studies in line with the terms used in the papers; generally, the authors did not report how they assessed sex/gender/gender identity

### Study Findings

The key findings of each study can be found in Table 4, and the findings are also summarized below.

#### Discrimination and Victimization

Many of the studies explored victimization-related mediators. Perceived or actual sexual orientation-specific discrimination was found to be a mediator in three American adolescent samples, while peer victimization was found to be a mediator in longitudinal studies with young people in the UK and the Netherlands. Bias-based victimization was also demonstrated to be a mediator in a sample of Black American youth. Cyberbullying victimization was found to be a significant mediation in a longitudinal youth sample and in a Black American youth sample. Furthermore, victimization and daily discrimination attenuated the relation between same-sex contact and depressive symptoms in a cross-sectional young adult sample.

One of the studies found that incivility and heterosexist harassment mediated the relation between sexual minority status and depression in university students. However, in another university sample, the association between sexual minority status and depression was mediated by harassment due to gender nonconformity but not harassment due to sexual minority status. Furthermore, in two studies that controlled for additional factors, the role of victimization as a mediator was reduced: perceived and hate-crime victimization attenuated the association between sexual orientation and depression in a Swedish adult sample; however, when controlling for familial confounding, depression differences between heterosexual and sexual minority participants were smaller, albeit still statistically significant for women. Similarly, the effect of sexual minority status on depressive symptoms was largely attenuated when controlling for unmeasured familial confounding by comparing sexual minority youth to their heterosexual same sex twin siblings in another Swedish study. Adding general victimization in the model had limited impact on the association.

*Conclusions* Overall, there has been consistent evidence from many adolescent and young adult populations from several countries suggesting that discrimination and victimization variables mediate the relation between sexual orientation and depression. However, there has also been some evidence that adjusting for confounder variables such as familial confounding reduces the mediated effects.

#### Physical or Sexual Violence

Physical and sexual violence and abuse were investigated in some studies using assessments that did not specify whether participants thought that these experiences were due to their sexual orientation. Dating violence was found to be a mediator in the relation between sexual orientation and depression in a sample of Chinese university students. History of abuse was found to be a significant mediator in a sample of Australian women. An aggregate adversity measure that included physical and sexual abuse in childhood, housing adversity, and intimate partner violence mediated the relation between sexual orientation and various mental health outcomes including depression in an U.S. youth sample. In a female adult sample, sexual and physical abuse and parental neglect prior to age 18 were found to mediate the association between sexual minority status and depressive symptoms only when comparing heterosexual to bisexual women; no depression differences were found between heterosexual and lesbian women. Similarly, another study found history of sexual trauma was one of the factors mediating the association between bisexual identity and depression, while gay and lesbian identities did not predict depression.

One included study reported some conflicting findings. Lesbian or bisexual identities were not significant predictors of depression, but identifying as mainly heterosexual was significantly associated with depression. Mainly heterosexual women still reported higher rates of depression than heterosexual women after controlling for interpersonal violence, suggesting that there was not sufficient evidence of interpersonal violence as a mediator, despite interpersonal violence being a predictor of depression in the model.

*Conclusions* Physical and sexual abuse was found to mediate the relationship between sexual orientation and depressive outcomes in most studies that tested such variables, particularly for individuals with a bisexual identity.

#### Stress-related Factors

Studies reported positive findings when using a range of stress-related measures including perceived stress, a low number of positive events, social stress measures, negative life events and nonevent stress, in the form of barriers to core life pursuits. On the other hand, stress did not meet the criteria for mediation in a sample of university students once baseline depression was controlled for. In another study, perceived stress mediated the relation between sexual orientation and depressive symptoms when lesbian and bisexual women, mostly heterosexual women, discordant heterosexual women (i.e., women who identified as heterosexual but reported same-sex attractions and/or behaviors), and mostly heterosexual men were compared to concordant heterosexual men (i.e., men who identified as heterosexual and whose reported attractions and behaviors were all toward the opposite sex). However, this association was not found for gay, bisexual, or discordant heterosexual men, as these categories did not report more perceived stress than their heterosexual counterparts.

Unmet medical needs was found to be a significant mediating factor in the relation between sexual orientation and depression in a US youth sample. Physical health and health-related behaviors like smoking contributed to the relation between bisexual identity and depression, in a study that found depression disparities between heterosexuals and bisexuals but not between lesbian/gay and heterosexuals. Another included study found that time spent on cyber behaviors and social media was a significant mediator between bisexual attraction and depression outcomes, while such associations were not found when comparing gay and lesbian to heterosexual groups.

*Conclusions* Many studies suggested that different types of perceived stress were mediators, whilst a minority of studies showed some contradictory findings. Physical health disparities, unmet medical needs, and social media factors were also found to be mediators.

#### Family Relationships and Social Support

Studies investigating attachment and family-related factors had mainly positive findings with some evidence for sex and sexual orientation subtype differences. Cross-sectional samples of adolescents and young adults found secure attachment and family support, respectively, to attenuate the relation between sexual orientation and depression symptoms. However, conflicting evidence was found for attachment avoidance, which mediated the association between sexual minority status and depressive outcomes in an adult Israeli sample but was not supported as a mediator in a longitudinal adolescent sample. A study found some evidence of mediation for parental support but only when comparing bisexual to heterosexual women, as other groups were not found to differ in depression rates.

Gender or sex differences were found in several longitudinal studies. For example, parental rejection was found to be a significant mediator for girls but not for boys in a large longitudinal study. Similarly, another research study found that for girls, perceived closeness with parents, parental involvement, and perceived family support was a significant mediator, with perceived family support being the most important factor. For boys, family relationship variables seemed to explain less of the association than for girls, but closeness with parents was a significant mediator.

The relationships with mothers and fathers were also found to have a differentiating role in one of the studies: whereas lower relationship quality with fathers was a significant mediator in both men and women, lower relationship quality with mothers mediated that association only in women. On the other hand, reported family satisfaction mediated the relation between sexual orientation and later depression equally for girls and boys in one of the other included studies.

Evidence of mediation using general social support measures was reported in studies of adolescents, in women who had experienced intimate partner violence, and in bisexual versus heterosexual women who were survivors of sexual assault. Moreover, two other studies found that social support, along with other mediators, attenuated the relation between sexual minority identity and depressive symptoms and a third demonstrated the same finding but only for those with a bisexual identity. Social isolation was found to mediate the relation between same-sex attraction and depression in males but this was not found in females.

More systemic measures of social support were also found to be significant mediators: a sense of belonging in the community, negative perceptions of school environment, and institutional betrayal relating to sexual assault in undergraduate students.

*Conclusions* Studies generally indicate that sexual minority individuals experience to a lesser degree the protective effects of social support and other systemic factors (e.g., quality of the environment or a sense of belonging) and that these may help explain increased depression rates. Parental relationships and support may partly explain depression disparities, with some studies suggesting that this mechanism may be stronger for girls than boys. Internalized relationship representations and attachment styles may also play a role.

#### Intrapersonal Factors

A wide range of different intrapersonal psychological processes were investigated as potential mediators, with stronger evidence for self-esteem than for specific coping mechanisms. There was evidence from a few studies that self-concept, self-esteem, personal mastering, and a sense of mattering were significant mediators. Another study found evidence for mediation of a resilience measure that included items related to optimism and mastery. Lower emotional awareness and greater rumination were found to be significant mediators, but some of the studies did not find enough good evidence for other response styles, such as acceptance coping, denial, and blame.

Self-exploratory attitudes, including fun-seeking, number of sexual partners, and age of first sexual experience, did not account for the association between same-sex contact and symptoms of depression in a young adult sample. One study investigated sexual orientation concealment and found it to be a significant mediator in a sample of Chinese university students.

*Conclusions* Support for a range of intrapersonal psychological factors was reported, but each of these tended to be investigated in single studies and therefore require replication.

## Discussion

The aim of the present study was to review research evidence regarding psychosocial factors that may mediate the increased depression rates in sexual minority compared to heterosexual populations. Forty studies were identified and reviewed, examining as mediators constructs related to discrimination, victimization, violence, stress, social support and other interpersonal factors, as well as intrapersonal psychological processes.

It is perhaps unsurprising that such a diverse set of psychosocial factors have been proposed to explain the complex phenomenon of increased depression rates in sexual minorities compared to heterosexuals. The breadth of mediators suggested by existing evidence indicates that identifying the most important mediators is probably less crucial than recognizing the multitude of stressors that sexual minority individuals continue to face, and the different effects that such stressors have on individual psychological resources and coping mechanisms that make them either more vulnerable or resilient. Theoretical frameworks such as the minority stress model (Meyer, 2003) and the psychological mediation framework (Hatzenbuehler, 2009) help conceptualize the synergic effect of mediators and demonstrate the necessity of multiple and coordinated responses at different levels of the system. The implications for theory and intervention are further discussed in the next section.

It is worth noting that three of the studies reviewed controlled for familial confounding when examining victimization and maternal attachment as mediators. Two of these studies found that the mediation relations were weaker or disappeared when comparing among twin siblings. This led the researchers to suggest that shared genetic or environmental influences may play an important role in explaining depression disparities, without ruling out the possibility that minority stressors affecting heterosexual siblings may help explain their findings. In contrast, the third study found that attachment was still a mediator after controlling for sibling clustering. These findings along with other research (Zietsch et al., 2011) investigating shared etiological factors indicate that it is possible that genetic and/or environmental familial factors not directly related to sexual minority identification contribute to increased depressive symptoms in sexual minority individuals. It has also been argued that minority stressors and stigma may affect the heterosexual twins and other members of the family (Donahue et al., 2017; Timmins et al., 2018) which may help explain these findings.

### Implications

The vast majority of the studies demonstrate further evidence of increased prevalence of depressive symptomatology in sexual minorities in a diverse range of samples and age groups. This illustrates that sexual minorities continue to represent an at-risk population, reaffirming the importance of developing a comprehensive understanding of psychosocial processes that represent vulnerability factors. Such factors offer specific targets for preventative and therapeutic efforts.

The findings of this review are largely consistent with minority stress theory, according to which disproportionate stress related to stigma and discrimination results in elevated rates of psychological distress (Meyer, 2003). Most studies in this review report evidence that supports the suggestion that sexual minority individuals experience more stressors including harassment, victimization, violence, abuse, parental rejection, and other forms of adversity, and receive less social support and access to valued positive experiences than their heterosexual counterparts.

The review illustrates that there has been a greater emphasis in the literature on minority stressors than on the general interpersonal, emotional regulation, and cognitive processes through which such stressors increase people’s risk for depression (Hatzenbuehler, 2009). While understanding minority stressors is an important endeavor that can help address their impact on a broader sociopolitical and community level, a more careful exploration of these subsequent processes can provide targets for psychological interventions on an individual level. Where such factors were investigated, findings were consistent with Hatzenbuehler’s psychological mediation framework. Studies reviewed here reported low social support, increased rumination, low emotional regulation, poor sense of mastery, low resilience, and low self-esteem as factors that can help explain the increased rates of depression in sexual minority individuals. The literature could be expanded further to test all four components of Hatzenbuehler’s mediation paradigm. This can be achieved by using, for instance, serial mediation pathways to demonstrate how sexual minority orientation leads to increased exposure to stressors, which in turn lead to increased levels of individual psychological processes, which then contribute to elevated rates of depression. It is also important to gain a much better understanding of how different types of stressors may mediate depression risk via particular intermediate emotional/interpersonal/cognitive processes. Such specific pathways are not specified in Hatzenbuehler’s framework. An example would be to explore how experiences of family rejection based on sexual orientation lead to more negative beliefs about others and oneself that then put individuals at risk for depression.

These general psychological processes should be explored in parallel to group-specific proximal stressors such as internalized stigma, rejection sensitivity, and concealment that are also known to confer vulnerability for sexual minorities. For instance, the literature has suggested that dealing with issues of concealment is associated with maladaptive cognitive, affective, and behavioral strategies that are related to adverse mental health outcomes (Leleux-Labarge et al., 2015; Pachankis, 2007).

Policy makers, clinicians, families, schools, universities, and communities all have a role to play in addressing or mitigating the impact of stigma-related stressors for sexual minorities. Community, school, or university interventions that aim to target victimization, increase social support and benevolent experiences, and enhance positive identity development and a sense of belonging are likely to be protective. Moreover, evidence on parental and family support highlights the need for development of interventions that facilitate awareness, education, support, and normalization for parents and families, as well as access to support for youth who face family rejection or alienation due to concealed or disclosed identities.

Existing evidence about the cognitive and regulatory mechanisms that have been shown to be intermediate factors, both group-specific stressors such as internalized stigma and general processes such as lower self-esteem and rumination, can also inform the development of therapeutic interventions. Addressing such psychological processes is a key component of cognitive behavior therapy (CBT) for depression. There is emerging literature on CBT interventions adapted for sexual minority populations (e.g., Craig & Austin, 2016; Craig et al., 2012; Lucassen et al., 2015; Pachankis et al., 2015). Psychological interventions can also test psychological mediators as mechanisms of change in randomized-controlled trials (RCTs) for sexual minority individuals with mood difficulties. Change in both minority-specific and general potential mechanisms were investigated in an RCT that targeted minority-stress-focused processes in a transdiagnostic CBT treatment for sexual minority women experiencing depression, anxiety, and heavy alcohol use (Pachankis et al., 2020). The study provided evidence supporting the efficacy of the treatment in reducing participants’ depression and anxiety. They found no condition by time interactions for the minority stress processes (rejection sensitivity, concealment, internalized stigma) or general processes (emotional regulation difficulties, rumination, and assertiveness) with the exception of social support which showed results in the opposite direction to expected. However, in pooled analyses, they found small-to-medium pre–post-reductions for the general processes with small effects for the minority stress processes. Future minority-specific interventions may consider addressing other processes such as those arising from the increased levels of violence and abuse experienced by sexual minority individuals (Roberts et al., 2010). Finally, although more research is needed, mental health practitioners can use this review as a guide of the multitude of vulnerability factors that can be considered in case formulations and interventions with sexual minority clients presenting with depression.

### Limitations of Studies

The methodological quality of studies varied, with just under a fifth of the studies suffering from important methodological limitations. Many studies had significant response and attrition issues. Furthermore, only one study used diagnostic interview questions to assess the presence or absence of clinical depression, rather than relying on self-report measures of depressive symptoms or the presence of a depression diagnosis. While self-report questionnaires can indicate high levels of depressive symptoms, they should not be used on their own to diagnose depression. Psychiatric diagnosis requires that the individual has a minimum number of a set of symptoms, experiences them at a specified frequency, suffers significant impairment in at least one life domain as a result of the symptoms, and that other possible causes of the symptoms have been excluded. In addition, many studies used measures for their mediators for which there was inadequate evidence of validity and/or reliability.

Another serious methodological issue was that the majority of the reviewed studies were cross sectional. A cross-sectional design does not allow the examination of causal pathways and therefore conclusions cannot be drawn about the predictive value of the independent variable and the mediators. For example, one might argue for reverse causality, arguing that, for instance, depression may lead to isolation and decreased social support. Furthermore, many studies used retrospective self-reports to assess mediators such as victimization, abuse, and social support. When retrospective self-reports are used, recall biases may inflate the associations demonstrated. This is especially the case if mediation measures are collected at the same time with measures of depression, as the mood-congruent memory bias observed in depression (e.g., Watkins et al., 1996) could affect the way individuals report their past experiences.

Another complication related to temporality was that a few cross-sectional studies used history of experiences such as victimization without specifying a specific time frame. Such experiences might have therefore occurred before participants identified as sexual minority (e.g., in childhood). This violates mediation theory in that the independent variable would not necessarily precede the mediator in time. It is hence debatable whether these studies can claim that they provide evidence for mediation.

While some of the studies measured variables of interest in a longitudinal fashion, none of them used data from several different waves of measurement; they either measured sexual orientation and mediator at time 1 and depression at time 2, or sexual orientation at time 1 and mediator and depression at time 2. In addition, some of the longitudinal studies did not control for baseline depression levels and none of the studies controlled for baseline measures of the mediator. Controlling for baseline scores is important as it generally explains a great deal of the variance in later measures, thus improving precision and power to detect effects of the mediator on the outcome.

Many studies had significant limitations in the statistical approaches they used to examine mediation. Many used the causal steps approach (Baron & Kenny, 1986) without then calculating an indirect/mediated effect or conducting a statistical test for this effect. The limitations of using the causal steps approach without examining the mediated effect have been well documented in mediation analysis literature (e.g., MacKinnon et al., 2002). Whereas it is important to demonstrate that sexual minority status is related to mediators (a path) and mediators are related to depressive outcomes (b path), if one is conducting mediation analysis the magnitude of the mediated effect is also of interest, which can only be examined if an indirect effect is calculated and evaluated with a test of significance. Moreover, many studies did not report exactly which statistical tests they used. Others failed to follow good practices in reporting mediation analysis results, such as reporting which tests they used for testing the significance of the indirect effect, or presenting confidence intervals of direct and indirect paths. In addition, although many studies had large samples, very few studies provided a justification for sample size selected or reported a power analysis. One study did not report estimates for individual mediators which makes interpretations about their unique contribution very difficult. Finally, the lack of reported effect sizes by many studies and the diverse statistical methodologies used precludes us from being able to usefully comment on or compare between mediation effect sizes in different studies. Even when studies used the same constructs as mediators (e.g., victimization), they operationalized and assessed them in different ways. Therefore, drawing conclusions about the comparative strength or importance of mediators between studies would be misleading. It would be useful to have a unified method for reporting mediation studies so that effect sizes could be extracted and meta-mediation analyses could be conducted.

A significant minority of the studies used large cohort samples which are generally more representative of the population they wish to test. However, many of the studies used subsamples of the population or convenience samples. For example, the wide use of university samples by the studies, while common in psychology research, has been argued to be problematic as university students tend to have higher socioeconomic status and are largely homogenous (Hanel & Vione, 2016). Moreover, over a fourth of the studies included in this review used the same or overlapping samples, albeit using different designs and time points and testing different mediators.

There was considerable variability in how sexual minority status was defined and categorized, as well as to how depression was measured, which limits the ability to directly compare results across different studies in reviews and meta-analyses. An ongoing issue in sexual minority research is the operationalization and measurement of sexual minority status. Definitions can be based on identity, behavior, attraction, and/or preference for romantic partners; a few studies use combinations of these factors. Future research should use multiple indicators and investigate, for example, whether the implications of self-labeling as a sexual minority, and therefore associating oneself with a stigmatized identity, are different in relation to depression than same-sex behaviors in the absence of such an identity.

Furthermore, most studies did not investigate differences across sexual minority subgroups due to issues with sample size and power, with some of the studies ignoring or excluding some groups from their analysis. Further research can aim to develop a better understanding of the distinct issues and outcomes that different sexual minority groups face. For example, there is some evidence to suggest that bisexuals may have especially high risk for mental health problems (Burns et al., 2016; Hughes et al., 2014; Luk et al., 2019; Needham & Austin, 2010). Groups identifying as mostly heterosexual are also poorly understood with some research included in this review reporting similar or worse outcomes for them compared to other sexual minority groups (Corliss et al., 2009).

In this review, terms such as “boys,” “girls,” “men,” and “women” are used to report the findings of studies in line with the terms used in the papers; generally, the authors did not report how they assessed sex or gender. In most studies, gender identity was not assessed or discussed, and the distinctions between sex, gender, and gender identity were not taken into account. Future studies should explore the gender identity of participants and report how their gender or sex was assessed.

The overwhelming majority of studies reviewed took place in specific locations within the U.S. Research findings will be affected by the policy and societal climate of the time and place in which the studies were conducted. Sexual minority stressors are likely to vary significantly across countries as well as within countries in different sections of the population. As research continues to take place in other locations, it will be important to compare the factors mediating depression risk for sexual minorities across different social contexts.

### Limitations of the Review Process

This review did not include grey literature and research that was not published in peer-reviewed journals. As a consequence, it is possible that the well-documented bias of reporting and publishing mostly positive results in scientific journals can affect the conclusions. Although this review did present a few negative findings, it is still likely that a publication bias conceals research findings about factors that do not mediate the differing depression rates among sexual minority and heterosexual individuals. Moreover, this review did not include research that was not published in languages other than English which may have restricted the inclusion of studies that took place in different parts of the world. Lastly, as aforementioned, the heterogeneity in methodological approaches among studies, including the use of different operational definitions of sexual minority orientation and depression, undermines comparisons and synthesis of findings between studies. The overview of the findings in the Results section is open to authors’ biases but readers can find much more detailed information in the tables and the Appendix.

### Conclusions and Directions for Future Research

This review found evidence consistent with suggestions that stressors such as victimization, harassment, abuse, life stress, and reduced social and familial support contribute to the increased depression rates found in sexual minority individuals compared to heterosexuals. There was also some evidence suggesting that differences in psychological processes such as self-esteem, mastery, emotion regulation, rumination, and coping styles may also play a role. Such understanding is important in directing wider sociopolitical factors and policy issues that can help address mental health inequalities, as well as informing community and clinical interventions.

However, the methodological limitations of the studies mean that no firm conclusions should be drawn and higher quality research is needed. Prospective studies are required in which sexual orientation, mediators, and depression outcomes are assessed at three consecutive time points. Furthermore, appropriate statistical methods should be used to examine mediation processes. Studies using longitudinal designs with at least three time points to test mediators of sexual minority status and depression would allow researchers to draw firmer conclusions about which mediators should be the target of interventions. Randomized research designs could then be used in which at-risk individuals are offered interventions aimed at addressing a hypothesized mediator, such as self-esteem or social support. Proper estimation of mediated effects in intervention studies would provide the best test about the impact of hypothesized mediators in the depressive symptomatology of sexual minority individuals by assessing whether the intervention targeted the mediator of interest and whether the mediator of interest had in turn an effect on depressive outcomes. Studies using structured assessment of clinical depression are required to overcome some of the limitations associated with self-report of depressive symptomatology.

Since this review was conducted, we have published a study that addressed many of these methodological issues outlined here, by assessing variables at three separate time-points, controlling for baseline depression, and using robust statistical methodology in a large longitudinal sample of UK youth. The study found evidence of poorer family relationships and unhelpful assumptions as mediators as well as weaker support for self-esteem as a mediator (Argyriou et al., 2020).

Further research is needed to better understand possible psychological mechanisms through which minority stressors exert their impact on mental health. Research could also explore whether and how shared genetic or environmental factors relate to the elevated depression risk of sexual minorities, independently from minority stressors. Different aspects of sexuality such as attraction, identity, and behavior should be studied in order to better understand their association with different risk factors and outcomes. Some studies in this review reported distinct findings for males and females, but more research into differences in relation to sex and gender identity is required. Greater use of general population samples rather than convenience samples would help overcome potential issues of participation bias. However, where specific subgroups have been underrepresented (e.g., bisexual or mostly heterosexual individuals), targeted recruitment methods may be required. Finally, further research is needed from other countries and cultures and across sexual minority subgroups, including bisexual and mostly heterosexual individuals, as processes may vary between individuals experiencing different types of minority stressors.

## Appendix

**Table:**

Study	Sample characteristics	Measures for mediator(s)
1. Almeida et al. (2009)	Age: 13–19 years (*M* = 16.3, *SD* = 1.3) Gender: 58.3% females, 41.7% males Ethnicity: 30.7% Hispanic, 44.8% non-Hispanic Black, 10.8% Asian/Pacific Islander/biracial/multiracial/other	Perceived discrimination: single yes/no item
2. Burns et al. (2016)	Age: 48.5% of the sample were between 20 and 24 years, 51.5% of the sample were 40–44 years Gender: 47.9% females, 52.1% males	Major life events: Age first moved away from the parental home; age first moved in with first partner; age of first sex; measure of traumatic life events Social support: Estimated from a factor analysis of the *Schuster Social Support Scale* (Schuster, Kessler, & Aseltine, 1990) assessing family, partner, and friend negative/positive support Health and behaviors: Continuous Short Form-12 Physical Health Component Score that was computed following the RAND scoring (Hays, Sherbourne, & Mazel, 1993), and a binary indicator for Self-Rated Health Behavioral activation and inhibition: *BIS*–*BAS*, a measure of behavioral activation and inhibition (Carver & White, 1994)
3. Burton et al. (2013)	Age: 14–19 years (*M* = 17, *SD* = 1.36) Gender: 70% females, 20% males Ethnicity: 31% White, 63% African-American, 3% other	Sexual-minority specific victimization: Victimization due to actual or perceived sexual minority status assessed via four items
4. Donahue et al. (2017)	Age: 18 years Gender: 59.3% females, 40.7% males	Victimization: Dichotomous variable based on reports of experiencing emotional abuse, physical abuse/neglect, sexual abuse/assault
5. Frisell et al. (2009)	Age: 20 to 47 years (*M* = 33.7) Gender: 39.8% females, 60.2 males Education: 4.4% low, 47.3% medium, 45.7% high, 2.6% missing Currently in relationship: 73.5% yes, 25.3% no, 1.2% missing	Perceived victimization: Self-report of ever having been “discriminated against in an insulting or disparaging way.” Hate crime victimization: Single item asking whether respondents have experienced violence due to their “race, ethnicity, gender, sexual orientation, or religion”
6. Frost and LeBlanc (2014)	Age: *M* = 31.71 years (*SD* = 10.75) Gender: 69.4% females, 30.6% males Ethnicity: 71% White, 14% Black, 6% Latino, 6% Asian, 2% Native American, 1% Pacific Islander, 7% other Education: 57% some college or more, 43% high school diploma or less Employment: 52 full-time, 20 part-time, 14 unemployed, 36 student Relationship status: 43% single, 57% in a relationship	Nonevent stress: assessed in the form of perceived barriers to participants’ pursuit and achievement of personal projects. Barriers to project pursuit were measured with the *Personal Project Inventory* (PPI; Little, 1983) (*α* = .58 to .90)
7. Hatzenbuehler et al. (2008)	Age: 11–14 years Grades: 31.8% in sixth grade, 33.9% in seventh grade, 34.3% in eighth grade Gender: 48.8% females, 51.2% males Ethnicity: 13.2% non-Hispanic White, 11.8% non-Hispanic Black, 56.9% Hispanic, 2.2% Asian/Pacific Islander, 0.2% Native American, 0.8% Middle Eastern, 9.3% biracial or multiracial, 4.2% members of other racial/ethnic groups, 1.3% unspecified racial/ethnic background Household: 27.4% lived in single-parent households	Emotional regulation: emotional awareness and rumination: Emotional awareness subscale of the *Emotion Expression Scale for Children* (Penza-Clyve & Zeman, 2002) assesses extrinsic processes of emotion regulation. (*α* = .88 for full sample; *α* = .87 for heterosexuals; *α* = .91 for sexual minorities) *Children’s Response Styles Questionnaire* (CRSQ, Nolen-Hoeksema & Morrow, 1991) (*α* = .86 for full sample; *α* = .86 for heterosexuals; *α* = .81 for sexual minorities)
8. Hatzenbuehler et al. (2012)	Age: 12–18 years (Grades 7 to 12) Gender: 51% females, 49% males	Social network variables based on peer nominations with 3 indicators: Social isolation: Two measures of social isolation were calculated: (a) in-degree (b) out-degree Degree of connectedness: The total number of students the participant could reach in three steps in the participant’s network Social status: Bonacich’s (1987) centrality measure was used to capture social status within the peer network
9. Hughes et al. (2014)	Age: *M* = 45.18 years (*SE* = 1.21) Ethnicity: 65.3% non-Hispanic White, 20% non-Hispanic Black, 11.6% Hispanic, 3.1% other Education: 37% high school or less, 31.9% some college, 16.8% college degree, 14.2% graduate/professional degree Residence: 59.9% urban, 15.7% rural, 24.4% Chicago metropolitan	Victimization: A measure of cumulative victimization that summed the number of types of victimization experienced across the lifespan was created including different types of childhood abuse and adult victimization and intimate partner violence.
10. Krueger et al. (2018)	Age: 24–34 years Gender: 53.3% females, 46.7% males Ethnicity: ~ 11.8% Hispanic, ~ 15.7% non-Hispanic Black, 3.25% non-Hispanic Asian, ~ 69.1% non-Hispanic White	Perceived stress: Four-item version of the Cohen Perceived Stress Scale (Cohen, Kamarck, & Mermelstein, 1983)
11. la Roi et al. (2016)	Age: wave 1: *M* = 11.1; wave 2: *M* = 13.6; wave 3: *M* = 16.3; wave 4: *M* = 19.1; wave 5: *M* = 22.3 Gender: 54.8% females, 45.2% males	Peer victimization: Single item on bullying and three items on relational victimization (*α* = .85) Parental rejection: Self-reported parental rejection from the *EMBU*-*C* (Markus, Lindhout, Boer, Hoogendijk, & Arrindell, 2003). wave 1: *α* = .84 for rejection by father; *α* = .84 for rejection by mother; wave 4: *α* = .70 for rejection by father; *α* = .67 for rejection by mother
12. Luk et al. (2018)	Age: From 11th grade to 3 years after high school, *M* = 17.2 at wave 2 Gender: 56.2% females, 43.8% males Ethnicity: 58.8% White, 17.3 African-American, 19.7% Hispanic, 4.3% other SES: 23.1% low, 50% middle, 27% high	Family satisfaction: Single item on self-reported satisfaction with the relationships in their families. Responses coded as low, moderate, high, or very high Peer support: Participants nominated up to 6 of their closest male and female friends and the indicated whether they have talked with each of the friends about a problem in the last week Cyberbullying victimization: Single item on cyberbullying Unmet medical needs: Single yes/no item
13. Luk et al. (2019)	Age: wave 2: *M* = 17.2, *SD* = 0.51; wave 7: *M* = 22.6, *SD* = 0.53 Gender: 59.4% females, 40.6% males Ethnicity: 58.9% White, 19.7% African-American, 17.2% Hispanic, 4.3% other Family affluence: 23.1% low, 49.7% medium, 27.2% high	Cyber behaviors: Two items (weekday, weekend time) assessed the number of hours per day participants usually use a computer, the Internet, or a cell phone for chatting online, e-mailing, texting, tweeting, or social networking. Final items ranged from 0 (none at all) to 7 (about 7 or more hours a day) Social media use: Participants reported frequency of engagement in seven different activities on a social networking site in the past three months. Response options ranged from 0 (never) to 5 (multiple times a day) (*α* = .91)
14. Martin-Storey and August (2016)	Age: non-LGB *M* = 20.3 years (*SD* = 1.5), LGB *M* = 20.29 years (*SD* = 1.4) Gender: 58% females, 42% males Ethnicity: 19.5% Asian, 4.4% Black/African-American, 61% White, 10% Hispanic, 5% unspecified Family-of-origin income: Incomes were distributed over the range presented, with 7.2% of the sample having families with incomes of $20,000 yearly or less Residence: 36% grew up in a city, 53% in a suburb, 11% in a rural area Education: 91% attended a university, 9% attended a community college	Harassment due to gender nonconformity: Frequency with which had experienced victimization events “because other people think that the way they act or dress does not match the sex they were assigned at birth, or their gender nonconformity” (*α* = .84) Harassment due to sexual minority status: Frequency with which had experienced victimization events “because of their actual sexual orientation or their perceived sexual orientation.” (*α* = .82)
15. Martin-Storey and Crosnoe (2012)	Age: 15 years Gender: 50.9% females, 49.1% males Ethnicity: 81% White, 12% African-American, 1% Asian or Pacific Islander, 5% other Family structure: 63% had father present at home	Harassment due to sexual minority status: Experiences of harassment in the past year because of their sexual orientation Self-concept: Identity subscale of the *Psychosocial Maturity Inventory* (Greenberger, 1976) includes questions addressing self-esteem and coherence of self-concept (*α* = .77; *α* = .84 for sexual minority youth) Self-regulation: both primary caregiver’s and youths’ reported self-control from the subscale of the *Social Skills Rating System* (Gresham & Elliot, 1990) (Maternal *α* = .83, .87; Adolescent *α* = .74, .68) Friendship quality: Perception of friendship quality with a best friend based on *Friendship Quality Questionnaire* (Parker & Asher, 1993) (*α* = .92; .85 for sexual minority youth) Parental support: Scale assessing the youth’s perceptions of their primary caregiver’s caring and attentive behavior, with higher scores reflecting greater maternal warmth (*α* = .92; .90 for sexual minority youth) Quality of the school environment: Latent factor that combined school attachment (*α* = .76), teacher bonding (*α* = .61), and negative attitudes toward school (*α* = .69). The subscales were drawn from the What My School is Like Questionnaire, adapted from the New Hope Study (Duncan, Huston, & Weisner, 2007)
16. McLaren (2008)	Relationship status: 55% heterosexuals/57% lesbians were married or in a committed relationship Education: 48% of heterosexual women and 29% of lesbians had completed secondary school; 25% of the heterosexual women, and 47% of the lesbians had completed a university degree	Sense of belonging: The Psychological subscale of the *Sense of Belonging Instrument* (Hagerty & Patusky, 1995) *α* = .94 for heterosexuals; *α* = .94 for lesbians
17. McLaren et al. (2007)	Age: *M* = 39.02 Relationship status: 41% gay men/23% heterosexual men were single, 48% gay men/68% heterosexual men were married or in a committed relationship, 10% gay men/9% heterosexual men were separated or divorced, and 1% of gay men/0% of heterosexual men were widowed Education: 64% gay men/51% heterosexual men had a university degree Residence: 73% gay men/63% heterosexual men leaved in an urban setting; 27% gay men/37% heterosexual men leaved in a rural setting Income: heterosexual men had a higher average income than gay men	Sense of belonging: The Psychological subscale of the *Sense of Belonging Instrument* (Hagerty & Patusky, 1995) *α* = .95 heterosexuals; *α* = .96 gay males
18. McLaughlin et al. (2012)	Age: 18–27 years Gender: 47% females, 53% males Ethnicity: 66% non-Hispanic White, 16% non-Hispanic Black, 12% Hispanic, 7% other Education: 52% enrolled or completed college, 48% no college	Exposure to adversity: Aggregate dichotomous variable based on whether respondents scored positively on any of the following: Single item on childhood physical abuse by caregivers; single item on childhood sexual abuse from caregivers; 2 items assessing housing-related adversity; 3 items on intimate partner violence
19. McNair et al. (2005)	Two subsamples included: younger cohort/older cohort Age: 22–27 (younger cohort), 50–55 years (mid-age cohort) Residence: random sampling from Australian population register; oversampling from rural and remote areas	Stress: The *Perceived Stress Questionnaire for Younger Women* (PSQYW, Bell & Lee, 2002) assesses self-reported stress on 10 items from five life domains Abuse: Single item on childhood or adulthood physical, mental, emotional or sexual abuse or violence Social support: The degree of social support was assessed by a modified version of the *Medical Outcomes Study (MOS) Social Support Scale* (Sherbourne & Stewart, 1991). Respondents were asked “How often is each of the following kinds of support available to you if you need it?”, with six items to assess social support in five dimensions: emotional support, information support, tangible support, positive social interaction and affectionate support
20. Mereish et al. (2019)	Age: 10–18 years (26.2% in Grade 6, 21.6% in Grade 8, 27.8% in Grade 10, 22.1% in Grade 12) Gender: 51.7% female, 47.2% male	Cyber victimization: assessed with one item: “During the past 12 months, how often have you been electronically bullied by someone?” Bias-based victimization: assessed with single item: “During the past 12 months, how often were you bullied for any of the following reasons? race, ethnicity, or national origin; religion; gender; because you are gay, lesbian, or bisexual, or someone thought you were; a physical or mental disability; because of your language or accent; and any other reason (*α*. 0.71)
21. Miller and Irvin (2016)	Age: *M* = 46.6 Gender: 78% females, 22% males Ethnicity: 71% White; 11% Black; 3% Hawaiian/Asian; 2% Native American; 4% Hispanic; 8% multiracial; 1% other Income: 26% low; 40% medium; 15% medium high; 19% high Education: 9% less than high school; 28% high school graduate; 32% some college; 30% college graduate	Type of victimization: Measured with three items addressing sexual abuse by a partner, threats of physical abuse by a partner and physical abuse by a partner Emotional support: Single item: “How often do you get the social and emotional support you need?”
22. Needham and Austin (2010)	Age: 18–26 years at wave 3, *M* = 21.8 years Gender: 51% females, 49% males	Parental support: The measure combines respondents’ reports of maternal and paternal emotional support during young adulthood. Support is the sum of responses to three items for each parent: how close respondents feel to their parent, whether their parent is warm and loving, and whether they enjoy doing things with their parent. (*α* = .83 for the current residential mother support scale, *α* = .74 for the current residential father support scale, *α* = .86 for the previous residential mother support scale, and *α* = .89 for the previous residential father support scale)
23. Oginni et al. (2018)	Age: *M* = 25.95 Gender: All male sample Ethnicity: tribe: 77.45% Yoruba, 30.25% other Marital Status: Gay: 87.7% never married, 12.3% married. Heterosexual: 93.8% never married, 6.2% married	Family-related variables: included the marital status of the participants’ parents; a single question assessing the experience of neglect by parents in childhood; gender atypical behavior in childhood and the response of parents to it Resilience: assessed with the Positive Ideation subscale of the *Positive and Negative Suicide Ideation Inventory* (PANSI; Osman, Gutierrez, Kopper, Barrios, & Chiros, 1998). *α* = .77
24. Pakula et al. (2016)	Age: 27.5% 18–29 years, 22.5% 30–39 years, 25.7% 40–49 years, 24.4% 50–59 years Gender: 50.1% females, 49.9% males Education: 9.3% less than secondary school, 17.6% secondary school, 8.7% some post-secondary education, 64.3% post-secondary education Racialized minority: 21.9% yes, 78.1% no Marital status: 37.4% single/widowed/divorced, 62.6% married/common law Residence: 17% rural; 83% urban	Perceived life stress: Single item: “Thinking about the amount of stress in your life, would you say that most days are: not at all stressful, not very stressful or a bit stressful, quite a bit stressful, or extremely stressful?” Responses were recoded into a binary variable
25. Pearson and Wilkinson (2013)	Age: 12–18 years (Grades 7 to 12) *M* = 15.63 years Gender: 51.9% females, and 48.1% males Ethnicity: ~ 66% non-Latino White, 15% Black, 11% Latino, 3% Asian/Pacific Islander, 3% other	Family relationships: Items asking questions about each of the respondents’ relationship with their parents, e.g., “How close do you feel to your mother/father?”. Responses about mothers and fathers were combined by calculating the mean response to all 5 items for each parent (*α* = .84 for mother, *α* = .89 for father), and then took the mean of these two values for each respondent Perceived parental closeness: The number of the shared activities the respondent participated in with their mother and father in the past 4 weeks: (1) went shopping, (2) played a sport, (3) attended religious services or a church-related event, (4) went to a movie, play, museum, concert, or sports event, and (5) worked on a project for school Perceived family support: Five questions that asked respondents, “How much do you feel that: (1) your parents care about you? (2) people in your family understand you? (3) you want to leave home? (4) you and your family have fun together? (5) your family pays attention to you?” (*α* = .75)
26. Przedworski et al. (2015)	Age: *M* = 23.8 years Gender: 50% females, 50% males Ethnicity: 6.5% Black, 6% Hispanic, 14% East Asian, 10% South Asian, 63% White Relationship status: 46% not in a relationship, 37% in a non-cohabitating relationship, 3% engaged, 14% married or living together	Social stressors: Two items from the *Everyday Discrimination Scale* (Clark, Coleman, & Novak, 2004) were used (called names/insulted at least a few times a year; harassed or threatened at least a few times a year) and three items from the *UCLA Loneliness Scale* (Russell, 1996) (lack of companionship, feeling left out, and feeling isolated from others at least some of the time)
27. Riley et al. (2016)	Age: LGB *M* = 18.38 years, non-LGB *M* = 18.49 years Gender: 70.1% females, 29.9% males Ethnicity: LGB sample: 1.4% American Indian/Alaskan, 9.6% Asian, 2.7% Black/African-American, 11.0% Hispanic/Latino, 2.7% other, 1.4% Puerto Rican, 71.2% White Non-LGB sample: 0.5% American Indian/Alaskan, 11.6% Asian, 2.4% Black/African-American, 7.2% Hispanic/Latino, .5% multiracial, .4% Native Hawaiian/other, 2.8% other, 1.5% Puerto Rican, 72.1% White	Stress: *Perceived Stress Scale* (PSS; Cohen & Williamson, 1988) assessing experiences of stress; higher scores reflect greater cognitive appraisal of stressful life circumstances (*α* = .86) Coping styles: Brief *COPE* (Carver, 1997), assessing maladaptive and adaptive coping styles. Six scales did not yield adequate reliability and were not included in analyses. The scales included were: reframing (*α* = .72); institution seeking (*α* = .83); denial (*α* = .73); religion (*α* = .87); humor (*α* = .81); emotional support seeking (*α* = .75); substance use (*α* = .86); and blame (*α* = .71)
28. Robinson et al. (2013)	Age: 13–14 to 19–20 years Gender: 50.4% females, 39.6% males Ethnicity: Only “White British” sample was used	Victimization: Participants were asked whether they experienced specific forms of peer victimization during the previous 12 months. Parents also reported whether their child was bullied through name calling in wave 1 (no/yes).
29. Rosario et al. (2014)	Age: 17–25 years (*M* = 20.6, *SD* = 1.7) Gender: 64.4% females, 35.6% males Ethnicity: 93.9% White	Attachment: Scale assessing participants’ degree of satisfaction with their relationship with their mother across nine items (e.g., general communication, affection, support, respect, shared time, interests) (Jaccard & Dittus, 1991) (*α* = .94) Parental affection: Mothers reported their satisfaction with their relationship with their child across the same nine items completed by their children on the attachment measure (*α* = .94)
30. Safren and Heimberg (1999)	Age: 16–21 years, *M* = 18.2 Gender: 51.9% females, 48.1% males Ethnicity: LGB sample: 48% African-American, 2% Asian, 9% Hispanic, 32% White, 9% biracial, 2% Arabic Non-LGB sample: 58% African-American, 40% White, 2% biracial Education level: LGB sample *M* = 12.1 (*SD* = 1.5); non-LGB sample *M* = 12.4 (*SD* = 1.9) Living situation: LGB sample: 57% with parents; 16% with roommates; 11% other adult relative; 5% with grandparents; 5% with siblings; 4% on their own; 2% group or residential Non-LGB sample: 50% with parents; 10% with roommates; 11% other adult relative; 2% with grandparents; 6% with siblings; 2% in foster care; 2% group or residential	Social support: *Social Support Questionnaire* (SSQ; Sarason, Levine, Basham, & Sarason, 1983) yields scores for number of social supports, satisfaction with social support, and the degree to which a person perceives that he/she is satisfied with social support for help with problems (*α* = .98 and .96) Coping: The *COPE* (Carver, Scheier, & Weintraub, 1989) assesses how respondents deal with stressful situations. The factor thought to be more relevant to LGB adolescents, *coping through acceptance*, was selected that has items that assess coping by accepting one’s present circumstances, using restraint, and through positive reinterpretation and growth Stress: *Adolescent Perceived Events Scale* (Compas et al., 1987), respondents indicated the occurrence of stressful events in the past 4 months and how desirable or undesirable they were on a scale. Event ratings were categorized as negative or positive events, yielding total scores for each category
31. Shenkman et al. (2019)	Age: *M* = 28.46, *SD* = 5.36 Gender: 52.83% females, 47.17% males Relationships: 54.8% no partner Education: 61.2% had academic degree Place of residence: 88.4% lived in the city Place of birth: ~ 94% Israel, ~ 6% elsewhere SES: *M* = 3.30, *SD* = 0.94 self-rated economic status	Attachment avoidance was assessed on a subscale of the *Experiences in Close Relationships questionnaire* (ECR; Brennan, Clark, & Shaver, 1998). Participants rated the extent to which they agreed with each statement on a 7-point scale on 18 items (e.g., “I don’t feel comfortable opening up to other people in close relationships”) (*α* = .91)
32. Sigurvinsdottir and Ullman (2016)	Age: 18–71 years, *M* = 45.05 years Ethnicity: Heterosexual women: 48% African-American, 36% White, 2% Asian, 7.90% other, 13.10% Hispanic, 5.9% multiracial Bisexual women: 36.8% African-American, 41.1% White, 2.1% Asian, 10.6% other, 13.1% Hispanic, 9.5% multiracial Employment: 43.40% of heterosexual women and 37.9% of bisexual women were employed Education: Heterosexual women: 33.8% college degree or higher, 43.4% some college, 13.9% high school graduate, 8.9% not completed high school Bisexual women: 26.3% college degree or higher, 37.9% some college, 23.2% high school graduate, 12.6% not completed high school Income: Heterosexual women: 38.1% ≤ $10,000, 19% $10,000–20,000; 12.1% $20,000–30,000, 30.8% £30,000 Bisexual women: 47.3% $10,000 or less; 22.6% $10,000–20,000; 9.7% $20,000–30,000; 20.5% ≥ £30,000	Perceived social support: *Social Support Questionnaire* (SSQ; Sarason et al., 1983) (W1: *α* = .84, W2: *α* = .87, W3: *α* = .90) Frequency of social contact: 5 questions asking how often a person comes into contact with informal social network members (Donald & Ware, 1984). The composite score was based on the averaged items, with higher scores indicating greater frequency of social contact. (W1: *α* = .71, W2: *α* = .71, W3: *α* = .70)
33. Smith et al. (2016)	Age: 19–25 years Gender: 59.9% females, 39.8% males, 0.3% transgender-identified Ethnicity: 69% Caucasian, 11.2% Asian American/Pacific Islander, 7.7% Latino, 5.2% Black/African-American, 6.9% other	Institutional betrayal: A modified version of the *Institutional Betrayal Questionnaire* (IBQ; Smith & Freyd, 2013) measures institutional betrayal leading up to or after sexual assault. The instrument was given to participants who endorsed at least one at least one item on a sexual harassment and assault scale. Items include 7 questions about the role the institution played in the experience. Three additional items specifically examining the role of sexual orientation in institutional betrayal were added
34. Spencer and Patrick (2009)	Age: Mean 21.34 Gender: 69.6% females, 30.4% males Ethnicity: 88.2% non-Hispanic White, 11.8% other Living arrangement: 9.5% alone, 12.4% with a domestic partner, 40.5% with non-relatives, 37.9% with relatives/other Residence: 54.6% rural, 45.4% urban Relationship status: 55.2% in a committed relationship, 45.1% other Religion: 24.2% Protestant, 28.1% Catholic, 26.1% Jewish or other, 21.2% none Employment: 85.9% college/university; 14.4% employed/other	Social support: Measured with the *Medical Outcomes Study Social Support Survey* (MOS-SSS; Sherbourne & Stewart, 1991), which assesses several domains of social support including tangible support, emotional support, affective support, and positive support. Participants were asked how often each type of support was available to them if needed (*α* = .96) Personal mastery: The seven-item *Personal Mastery scale* (Pearlin & Schooler, 1978) assessing the consciously controlled cognitive-affective aspects of sense of control (*α* = .79)
35. Szalacha et al. (2017)	Age: 25–30 years Education: 10% year 10 or less; 19.2% year 12 or equivalent; 26.6% Trade/diploma; 34.2% university diploma; postgraduate degree 10.6% Income (AUD): 2% 15,999 or less; 4.9% 16,000–36,999; 11.6% 37,000–51,999; 81.1% 52,000 or greater Relationship Status: 34.7% single; 41.6% married; 20.4% De facto; 2.7% separated/divorced Parental Status: 68% no children; 32% 1 or more children Residence: 60.5% urban; 39.5% rural	Interpersonal violence: The *Composite Abuse* scale (Hegarty & Valpied, 2007). Participants were asked whether or not in the previous three years they had experienced: physical abuse; severe physical abuse; emotional abuse; sexual abuse; and harassment. An item assessed IPV. Responses to the items were summed to create a measure of interpersonal violence experiences
36. Tate and Patterson (2019)	Age: 24.25 – 34.67 (*M* = 28.98, *SD* = 1.75) Gender: 53.20% females, 46.80% males Ethnicity: 63.50% White/Caucasian, 22.8% African-American, 15.8% Hispanic, 8.8% other, 6.79% Asian descent, 3.4% American Indian Education: Ranged from 1 (eighth grade or less) to 13 (completed post-baccalaureate professional education) *M* = 5.68, *SD* = 2.19 Income: Annual income ranged from $0 to $1000 k (*M* = $35.36 k, *SD* = $45.11 k, Median = $30 k)	Perceived Stress: 4-item short form of the *Cohen Perceived Stress Scale* (Karam et al., 2012). Responses ranged from 0 (*never*) to 4 (*very often*), with higher scores reflecting higher perceived stress (*α* = .72) Parent relationship quality: assessed using questions about frequency of communication, quality of contact, and parental closeness. Responses were summed creating a composite score for each parent. Scores ranged from 3 to 15, with higher scores representing more favorable overall relationship quality (*α* = .73 for mother relationship quality; *α* = .79 for father relationship quality)
37. Teasdale and Bradley-Engen (2010)	Age: Average age = ~ 16 years Gender: 52% females, 48% males Race/ethnicity: 42% White, 20% African-Americans, 24% Hispanic, 14% other origins (including Asian, Native American and other) Location: 54% attended schools in suburban communities, 29% in urban communities, 17% in rural communities	Social stress: Measures of adolescent perceptions of prejudice by students (single item); witnessing/experiencing physical/sexual victimization experiences (3 items); family problems (single item about desire to run away from home); attempted/committed suicide of a close friend or family member were created Social support: Single item that asked respondents their level of agreement with the statement “You feel socially accepted.” Respondents were also asked how much they felt that parents, teachers, and friends care about them
38. Ueno (2010)	Age: 18–23 years (*M* = 20.02) Gender: 46% females, 54% males Ethnicity: 27.2% non-Hispanic White; 23.8 African-American; 24.8% Cuban; 23.7% other Hispanic; 0.1% other race Education: 80.4% graduated from high school	Victimization: *Inventory of traumatic events* (Turner and Lloyd, 2003) modified to focus on interpersonal coercion and violence Major discrimination events: total score from a five-item inventory (Williams, Yu, & Jackson, 1997) Daily discrimination: Items measuring minor but chronic and routine discrimination experience in daily life (Williams et al., 1997) (*α* = .85) Negative life events: measured by the total score from a 33-item checklist for a period of 12 months (Avison & Turner, 1988). Some of these items were also asked for partners and friends/relatives and added to each respondent’s total score Chronic strains: Wheaton’s (1994) measure modified to focus on life domains important for young adults: employment, school, residence, children, relationships with partners/parents, and general perceptions across domains Family support: Measured by a scale that focused on emotional support by family (Turner & Marino, 1994) (*α* = .91) Friend support: the summed score of eight items similar to family support items (*α* = .91) Optimism: *Life Orientation Test* (Scheier, Carver, & Bridges, 1994) (*α* = .67) Mastery: Pearlin and Schooler’s mastery scale (1978) (*α* = .73) Self-esteem: Rosenberg’s self-esteem (1965) scale (*α* = .78) Mattering: Summed score from a five-item scale (Rosenberg & McCullough, 1981) (*α* = .72) Fun-seeking orientation: The *Fun*-*Seeking Subscale of Behavioral Activation System* (BAS) (Gray, 1975) (*α* = .66) Relationship status: Dichotomous variable (1 = currently in a marital or dating relationship; 0 = otherwise) Number of sexual relationships: the lifetime total including opposite-sex and same-sex relationships Early first sex: Dichotomous variable (1 = had sex before age 15 for men or 16 for women; 0 = otherwise) Parents’/friends’ permissiveness of drug use: Summed scores from five-item scales (*α* = .70 for both parents’ permissiveness and friends’ permissiveness) Friends’ drug use: Summed score of a three-item scale (*α* = .78)
39. Wong et al. (2017)	Age: *M* = 20.9 years (SD = 3.7) Gender: 57.6% females, 39.3% males Education: 0.5% primary or below, 5.9% secondary, 91.8% tertiary or above. 93.7% of participants were university students Dating status: 73% dating, 3% cohabitating, 6.6% broke up in past month, 16.3% broke up in past year	Dating violence: The *Woman Abuse Screening Tool* (WAST; Brown, Lent, Schmidt, & Sas, 2000) assessing physical, psychological, and sexual violence at the most recent relationship Sexual orientation concealment: Two items assessing how many family and friends know about the respondents’ sexual orientation
40. Woodford et al. (2014)	Age: *M* = 23.1 years Gender: 61.2% females, 38.8% males Ethnicity: 72% White	Interpersonal mistreatment: Constructed measures assessing personal and ambient hostility, incivility, and heterosexist harassment. Respondents asked how often they had witnessed, heard, or knew about and personally experienced each behavior on campus in the past year. Each variable was dichotomized

Cronbach’s alphas (*α*) presented in this table are from the sample of the study in question
